# Measurement of Bacterial Headspaces by FT-IR Spectroscopy Reveals Distinct Volatile Organic Compound Signatures

**DOI:** 10.1021/acs.analchem.4c02899

**Published:** 2024-12-21

**Authors:** Christian Zenner, Lindsay J. Hall, Susmita Roy, Jürgen Hauer, Ronald Sroka, Kiran Sankar Maiti

**Affiliations:** † Technical University of Munich, School of Life Sciences, Intestinal Microbiome, Weihenstephaner Berg 3, 85354 Freising, Germany; ‡ University of Birmingham, Institute of Microbiology and Infection, Chair of Microbiome Research, B15 2TT Edgbaston Birmingham, U.K.; § Department of Clinical Medicine, Klinikum rechts der Isar, Technical University of Munich, School of Medicine and Health, Ismaninger Str. 22, 81675 Munich, Germany; ∥ TUM School of Natural Sciences, Department of Chemistry, Technical University of Munich, 85748 Garching, Germany; ⊥ Department of Urology, LMU University Hospital, LMU Munich, 81377 Munich, Germany; # Laser-Forschungslabor, LIFE-Center, LMU University Hospital, LMU Munich, 82152 Planegg, Germany

## Abstract

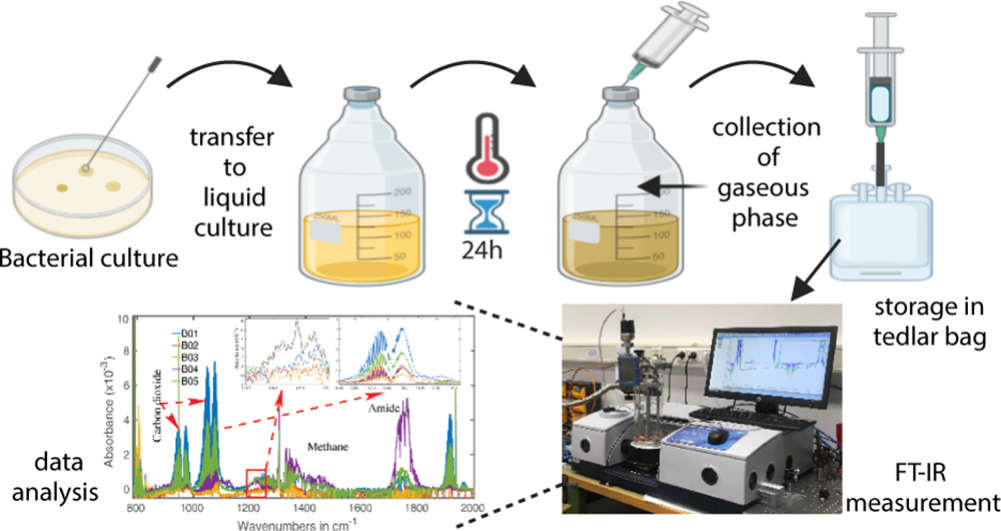

Ensuring prompt and precise identification of bacterial pathogens is essential for initiating appropriate antibiotic therapy and combating severe bacterial infections effectively. Traditional microbiological diagnostics, involving initial culturing and subsequent pathogen detection, are often laborious and time-consuming. Even though modern techniques such as Raman spectroscopy, MALDI-TOF, and 16S rRNA PCR have significantly expedited this process, new methods are required for the accurate and fast detection of bacterial pathogens. In this context, using bacterial metabolites for detection is promising as a future diagnostic approach. Fourier-transform infrared spectroscopy was employed in our study to analyze the biochemical composition of gas phases of bacterial isolates. We can characterize individual bacterial strains and identify specific bacteria within mixtures by utilizing volatile-metabolite-based infrared detection techniques. This approach enables rapid identification by discerning distinctive spectral features and intensities for different bacteria, offering new perspectives for bacterial pathogen diagnostics. This technique holds innovative potential to accelerate progress in the field, providing a faster and potentially more precise alternative to conventional diagnostic methods.

## 1 Introduction

Bacterial infections pose a significant threat to global health, contributing to many illnesses and fatalities annually. In 2019 alone, it was estimated that 7.7 million deaths were attributed to just 33 prevalent bacterial pathogens, highlighting the widespread impact of these infections on public health worldwide.^1^ More than half of these death cases were caused by only 5 bacteria: *Streptococcus pneumoniae*, *Staphylococcus aureus*, *Klebsiella pneumoniae*, *Escherichia coli*, and **Pseudomonas aeruginosa**.^1^ Regardless of the virulence of these bacteria, multidrug-resistant strains pose an even greater threat. The increasing global challenge of antimicrobial resistance necessitates prompt initiation of antimicrobial susceptibility testing to guide timely therapeutic interventions.^2,3^ Therefore, rapid and accurate identification is crucial to start with appropriate antibiotic treatment in order to save millions of lives.^4^ For sepsis cases, the current state-of-the-art approach for identifying bacteria in patients primarily relies on blood cultures to detect bacteremia.^5^ However, this culture-based diagnosis is not only a laborious and relatively slow process (at least 72 h) but is also plagued by several preanalytical limitations that can impact diagnostic performance. Issues such as inadequate blood volume collection, prior exposure to antibiotics, and delays in laboratory processing or transportation, particularly when facilities are off-site, significantly influence bacterial identification. Moreover, even if an organism is successfully cultured, definitive identification and susceptibility testing may be delayed by several days. Nowadays, whole-genome sequencing is frequently utilized in public health settings, but it is still relying on pure culture, sequencing, and analysis, not providing any time-saving compared to classical methods.^6^ Contamination at the blood culture (BC) collection stage is a common problem, leading to inappropriate antibiotic use, misguiding clinical diagnoses, and exposing patients to unnecessary toxicities. Additionally, standard automated systems may struggle with the cultivation of fastidious pathogens, further complicating the diagnostic process.^7,8^ Hence, it is essential to develop culture-free, rapid pathogen detection techniques.

Several emerging technologies are being developed to overcome the limitations associated with culture-based diagnosis. For instance, Raman spectroscopy-based imaging techniques promise rapid differentiation analysis between noninfectious systemic inflammatory response syndrome and sepsis.^9^ Additionally, feasibility studies involving endoscopic multicore fiber probes for remote scanning in clinical applications using such techniques are underway. These imaging techniques enable the identification of bacterial and fungal infections^10^ and also provide the opportunity to monitor treatment responses during infection and sepsis.^11,12^

Another promising technique for pathogen identification relies on microbial metabolites.^13−15^ It is well-known that all living organisms sustain themselves through various biochemical reactions, collectively termed metabolism.^16−18^ These metabolic processes yield distinct metabolites, characteristic of their respective pathways.^19^ Given the variation in metabolic processes among pathogens, analyzing bacterial metabolites offers a viable means of identification.^20,21^ Notably, volatile organic compounds (VOCs) derived from small metabolites play a pivotal role in pathogen diagnosis.^22−24^ The identification of bacterial VOCs predominantly relies on time-intensive analysis of the headspace of bacterial cultures. A more efficient approach is to identify bacterial VOCs directly from host biofluids, facilitating rapid diagnoses. While some researchers have explored breath analysis for this purpose,^25−27^ the full extent of bacterial metabolism and its interplay with host metabolism during infection remains incompletely understood.^28^ Achieving a comprehensive understanding necessitates a detailed exploration of the metabolic profiles of both bacterial hosts (in this case, humans) and the bacteria themselves.

To develop a basic understanding, it is imperative to employ a hierarchical investigative approach. Initially, a comprehensive examination of bacterial breath is essential for grasping the distinct volatile metabolic profiles of individual pathogens and differentiating them from those of collective pathogens. This task can be efficiently accomplished by analyzing the headspace of bacterial cultures, which calls for an appropriate analytical technique.

Various experimental techniques are available for metabolic analysis in the gas phase, including infrared spectroscopy,^29,30^ gas chromatography–mass spectrometry (GC-MS),^31,32^ electronic nose (e-nose),^33^ and laser spectroscopy-based multiwavelength UV photoacoustic methods.^34−37^ However, none of these techniques alone offer both high accuracy and cost-effectiveness for the unambiguous identification of metabolites. Among these methods, infrared spectroscopy-based identification of VOCs emerges as the most promising option due to its rapid and cost-effective analysis capabilities.^30,38−40^ It utilizes vibrations, a fundamental molecular property, to identify the molecule via structural analysis.^41−43^ Vibrational bands in the so-called fingerprint region of the spectrum represent a unique molecular or structural feature.^44^ Precise characterization of these molecular fingerprints, including their spectral position, intensity, and line shape, is crucial for advancing infrared spectroscopy in gas-phase biofluid analysis.^45,46^ In our research, we utilized infrared spectroscopy to identify individual bacterial pathogens and distinguish them when they are present as a mixture.

## 2 Methods

### 2.1 Bacterial Cultures

The following bacteria were selected for implementation and verification of sampling techniques and headspace measurements: **Escherichia coli** WS 1322 (B01), *Staphylococcus epidermidis* WS 4374 (B02), **Pseudomonas aeruginosa** DSM 19880 (B03), *Enterococcus faecalis* DSM 20371 (B04), and *Staphylococcus aureus* WS 228 (B05). Strains were provided from the Weihenstephan in-house culture collection.^47^

Bacteria were grown on trypticase soy yeast extract agar (DSMZ Medium 92; 30g/L trypticase soy broth; 3g/L yeast extract; 15g/L agar) at 37 °C for 24–48 h. Single colonies were restreaked three times to ensure the purity of the isolates. A single colony was transferred to 25 mL of trypticase soy yeast extract broth (TSYEB) in falcon tubes and incubated at 37 °C for 24–48 h. One milliliter of the bacterial culture was further transferred to 300 mL of TSYEB in 500 mL Schott bottles with a punch-through cap with a silicone septum and incubated for 24–48 h at 37 °C. Bacteria were cultured in triplicate. Negative controls were prepared by adding 1 mL of TSYEB to the culture bottles. A detail of bacterial cultures is presented in Table 1.

**Table 1:** Number of Replicates for Each Bacterial Species and Their Collection Dates

bacteria	no. of samples collected			
	29.06.2022	06.07.2022	28.07.2022	total
B01	2	1		3
B02	2	1		3
B03	2	1		3
B04	2	1		3
B05	2	2		4
Mix			4	4

### 2.2 Headspace Sampling

**Figure 1:**
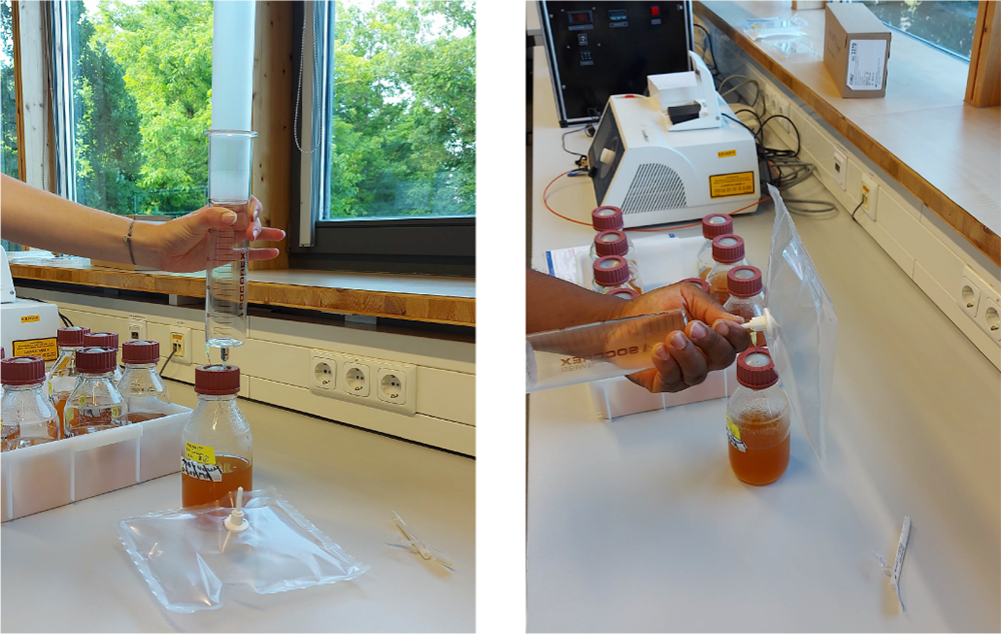
Process of headspace sampling. Left: Extraction of the gaseous phase from culture bottles was performed with a 250 mL glass syringe. Right: Filling of TEDLAR bags was used for storage of headspace samples.

To extract the headspace (bacterial breath), the silicone septum was punched with a 20G cannula attached to a 250 mL glass syringe. A second 20G cannula was punched through the septum to prevent the injection under pressure. The headspace of each culture bottle was pulled out four times to fill 1L TEDLAR bags used for gas sampling (Figure 1). All of the samples were collected at a time and sent for spectroscopic measurement immediately. All of the spectroscopic measurements were performed on the same day.

### 2.3 Sample Preparation for IR Spectra Measurements

**Figure 2:**
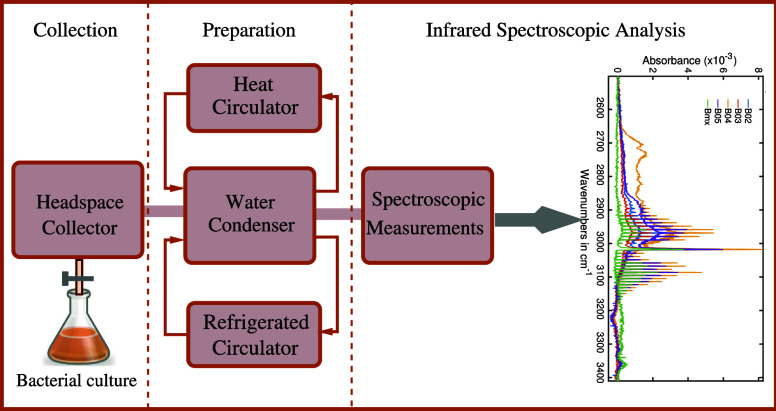
Schematic diagram of the experimental scheme for gaseous biofluid analysis by infrared spectroscopy. It consists of three major units: (1) Collection—In this part, headspace of bacterial culture is collected; (2) Preparation—water-suppressed samples are prepared; (3) Analysis—gaseous sample is collected in a multipass gas cell and measured with an FTIR spectrometer.

The primary obstacle in utilizing infrared spectroscopy for analyzing bacterial headspace is the significant presence of water vapor in the samples, which obscures the spectroscopic signature of bacterial metabolites due to its strong absorption in the infrared region.^46^ Recent advancements in water suppression techniques for gaseous biofluids have provided a promising solution for conducting infrared spectroscopic analysis on such samples.^48^ A custom-built water suppression system was employed to facilitate the infrared spectroscopic analysis. Figure 2 illustrates the schematic overview of this technique integrated with the spectroscopic measurement unit. Detailed information regarding the system and its operational principles has been previously documented.^48^ In essence, the sample preparation process involved two main components: (1) a sample collector and (2) a sample preparation unit.

The sample collector system was designed to accommodate both gaseous samples and the headspace of liquid biofluids. Before injecting the sample into the collector, the entire sample path was evacuated down to a pressure level of 10^–5^ mbar using two vacuum pumps, effectively eliminating any residual contamination from prior measurements. Bacterial breath samples were then introduced into the empty sample collector by releasing the valve.

The sample preparation unit consisted of a water condenser and both heat and refrigerated circulators. The water condenser was a sealed metal chamber housing a 12-m-long copper tube coiled into a spiral configuration, serving as the conduit for transferring the breath sample from the collector to a measurement cell. Before passage through the water condenser, the chamber was cooled to −60 °C using a refrigerated circulator. Once the condenser reached this temperature, the bacterial breath sample flowed through the copper tube at a controlled rate of 3 mL per second. During this transit through cold tubing, a significant amount of water vapor was effectively removed from the sample. An impressive water vapor reduction factor exceeding 2500 was achieved as the sample passed through the condenser at −60 °C. Subsequently, the water-suppressed gas-phase biofluid was transferred to the multipass sample cell. Following each experimental run, the copper tube underwent a cleaning procedure involving heating the chamber to 45 °C using a heat circulator and vacuum pumps.

### 2.4 Spectroscopic Measurements

All spectroscopic measurements of bacterial breath were performed using an FTIR spectrometer (Vertex 70, Bruker Optics GmbH, Germany). The spectrometer operated across a spectral range of 500–4000 cm^–1^ and employed a 4 m optical path length along with a 2 L “White cell” (Bruker Optics GmbH, Germany) for containing gaseous samples during spectroscopic analysis. To ensure consistency, one liter of headspace sample was used for each measurement. The spectrometer is purged with dry nitrogen to remove the water vapor from the spectrometer. Therefore, there is a gradual removal of water molecules over the time is expected from the spectrometer. To prevent significant differences in water vapor concentration between the background and sample scans, a background scan was conducted immediately before each sample scan. The absorption spectra of bacterial breath samples were recorded using a liquid nitrogen-cooled mercury cadmium telluride (MCT) detector. A spectral resolution of 0.5 cm^–1^ was maintained for all of the measurements. To minimize noise, 100 spectra were gathered and averaged for each sample. Acquiring 100 spectra typically takes about 5 min. In optimal conditions, each sample measurement, including preparation and system cleaning, requires around 20 min. The limit of detection (LOD) of the spectrometer is 10 parts per billion (ppb) for VOCs such as methane, acetone, carbon monoxide, etc., estimated using one liter sample of gaseous biofluid.^29^ It is important to note that for a given measurement system, the LOD is inversely proportional to the sample size. For instance, if the sample volume is reduced to one-fourth, the LOD increases four times.

### 2.5 Spectroscopic Data Analysis

The bacterial breath samples’ absorption spectra underwent component analysis utilizing the MATLAB programming language.^46^ Initially, the infrared spectra of the breath were scrutinized to identify significant spectral features. Subsequently, gas-phase molecular spectra were matched with the observed features in the breath samples using least-squares fitting to ascertain optimal agreement.^29^ Typically, gas-phase molecular spectra were sourced from commercial databases such as PNNL,^49^ HITRAN,^50^ and NIST44,^51^ or obtained experimentally and theoretically via quantum chemistry calculations.^29,52^

## 3 Results and Discussion

This study aimed to comprehensively analyze the metabolic profile of bacterial volatile metabolites, which we refer to as bacterial breath. To achieve this, five distinct bacterial strains were selected for cultivation. To ensure reliability, each strain was cultured in three separate sample bottles with one of them cultured four times. The findings from these experiments are detailed in subsequent sections.

**Figure 3:**
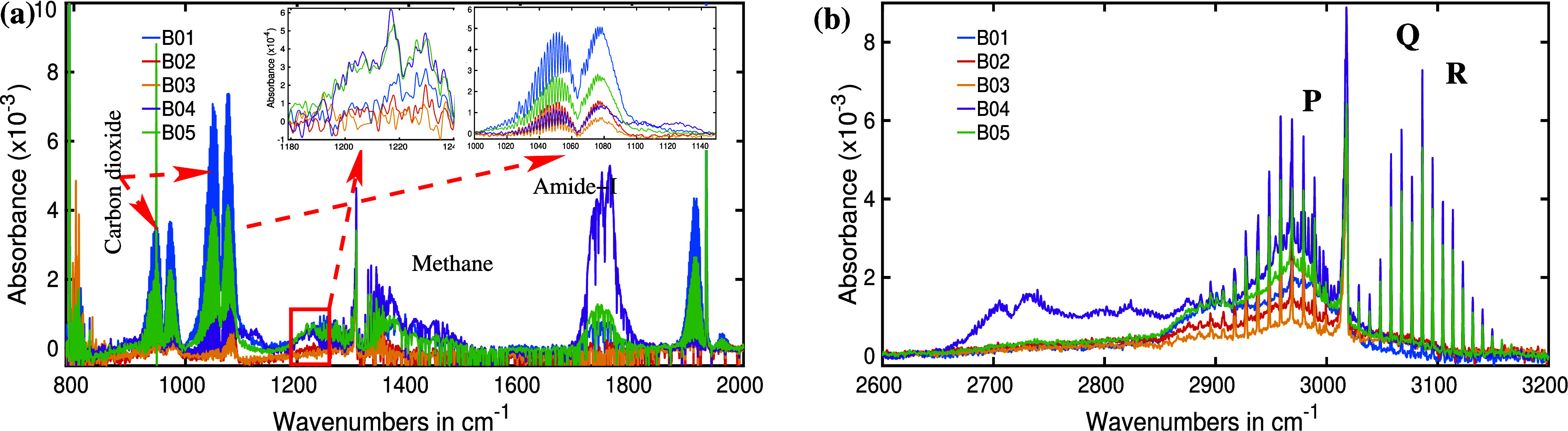
Infrared absorption spectra of the headspaces of five different bacteria. B01: *E. coli*, B02: *S. epidermidis*, B03: *P. aeruginosa*, B04: *E. faecalis*, and B05: *S. aureus*. (a) Several prominent spectral features are observed in the fingerprint region and attributed to bacterial metabolites. (b) Infrared absorption spectral region of methane for the bacterial headspace.

While the infrared spectra encompassed a broad spectral range, segment-wise spectra are presented for clarity.^53^Figure 3 highlights the two most significant spectral regions for all five bacteria. Each bacterial headspace spectrum (B01 to B04) represents the average of three measurements. *S. aureus* (B05) underwent four cultivation cycles; its spectra are an average of four spectral measurements.

Figure 3a,b reveals several distinct spectral features, each exhibiting variations in spectral positions and absorption intensities between the investigated bacterial species. For instance, Figure 3a displays noticeable peaks for carbon dioxide (CO_2_) at around 950, 1050, and 1900 cm^–1^. CO_2_ is a byproduct of various metabolic processes in living organisms. In this study, all bacteria examined exhibited robust CO_2_ absorption peaks in the measured headspace spectra, yet with significant differences in absorbance due to varying CO_2_ concentrations between the species. To illustrate, a magnified view of the CO_2_ peaks centered around 1050 and 1080 cm^–1^ is provided in the inset of Figure 3a. Among the bacteria studied, *P. aeruginosa* produced the least amount of CO_2_, while *S. epidermidis*, *S. aureus*, and *E. coli* produced approximately double, triple, and five times more CO_2_, respectively, compared to *P. aeruginosa*.

The bacterium *Enterococcus faecalis* (B04) exhibits a CO_2_ absorption peak 50% higher than that of *P. aeruginosa* (B03). B04 also yields an extremely high absorption peak at the amide-I band (1750 cm^–1^).^54^ Enterococci are frequently characterized as lactic acid-producing bacteria,^55^ making it highly likely that the amide-I band in the absorption spectra is attributed to lactic acid. However, a detailed chemical analysis is still required to confirm the specific molecular source of the amide-I band. Conversely, *P. aeruginosa* demonstrates no observable absorption at this band, suggesting a lack of gas-phase metabolites that would contribute to amide-I absorption in its infrared spectrum. Similarly, *S. epidermidis* (B02) also shows no discernible amide-I absorption peak.

An intriguing observation arises when comparing *E. coli* and *E. faecalis*: despite *E. coli* producing five times more CO_2_ than the latter, it yields only one-sixth of the absorbance at the amide-I band. *S. aureus* displays a moderate amide-I absorption, significantly lower than that of *E. faecalis*. It is worth mentioning that *S. aureus* provides a very strong absorption for CO_2_ compared to its amide-I absorption strength. Notably, these variations in absorption spectra provide insights into the metabolic profiles and chemical compositions of these bacterial species.

Figure 3b exhibits a similar trend. The predominant absorption spectra in this region come from methane, a common metabolite for the methanogenic bacteria.^56^ Methane is identified by its well-resolved **P**, **Q,** and **R** branches.^52^ The precise alignment of oscillations in all five absorption spectra unambiguously confirms the presence of methane in all selected bacteria. Among the tested bacteria, *E. faecalis* demonstrates the highest methane absorption peak, while *S. aureus*, exhibiting half the absorption strength of CO_2_ than *E. coli*, produces more methane. *P. aeruginosa* displays the lowest methane production among the selected bacteria. A broad absorption band is also observed at approximately 2725 cm^–1^, exclusively in *E. faecalis*. However, the molecular origin of this spectral feature remains unidentified at the moment.

**Figure 4:**
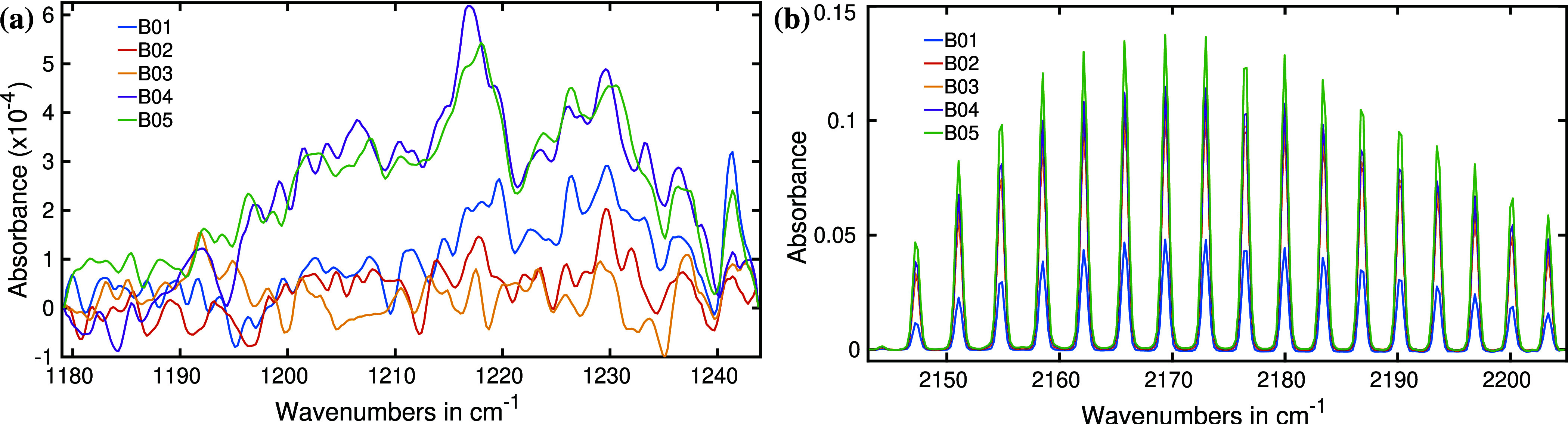
Zoomed infrared spectra of five bacterial headspaces in two different spectral regions. (a) Spectral fingerprint of acetone is observed for two out of five bacteria. (b) CO is identified for all five bacteria; however, its concentration varies significantly.

So far, spectral features that can be easily observed have been examined. However, numerous other spectral features with weak absorption also yield valuable insights for bacteria identification. The identification of those spectral features and their molecular assignments requires a rigorous spectral analysis using methods such as the “Matryoshka method”,^29^ ”multivariate curve resolution-alternating least-squares” (MCR-ALS)^57,58^ algorithm, and others. However, in this study, we limit our spectral analysis to visually distinguishable features, requiring only minimal analysis to establish bacterial identification through a VOC analysis. In a second step, we magnify different spectral regions in both the wavenumber and absorption axes, revealing a few more distinguishable spectral features. For instance, a pronounced peak is detected at approximately 1215 cm^–1^, as illustrated in Figure 4a. Noticeable distinctions in spectral characteristics emerge among the different bacteria. Both *E. faecalis* and *S. aureus* exhibit a shared feature with comparable spectral intensity, recognized as the molecular signature of acetone.^29^ The measured concentration of acetone is ∼440 ppb, much higher than the detection sensitivity of the instrument. Therefore, the assignment of this spectral feature is unambiguous. Conversely, *S. epidermidis* and *P. aeruginosa* are devoid of acetone spectral features. Although the presence of acetone in the headspace of *E. coli* is ambiguous, a distinctive spectral feature is evident at 1230 cm^–1^, yet its molecular origin remains to be determined.

A different spectral region (2140–2210 cm^–1^) is magnified and depicted in Figure 4b. The well-known spectral progression indicates the presence of carbon monoxide (CO). Among the five bacteria within this study group, all produce CO, although in varying quantities, which is reflected in the differing absorption intensities of the spectra. The measured concentration of CO varies from ∼5 to 15 ppm, which is 3 orders of magnitude higher than the detection limit. Therefore, the identification and assignment of this spectral feature are unambiguous. A notable observation in this spectral range is the behavior of *E. coli*, exhibiting the strongest absorption intensity for CO_2_, whereas yielding the lowest absorption spectra for CO among the selected bacterial group. *S. epidermidis*, *P. aeruginosa*, and *E. faecalis* exhibit similar CO production levels, while *S. aureus* demonstrates a higher amount of CO production in metabolic processes.

**Figure 5:**
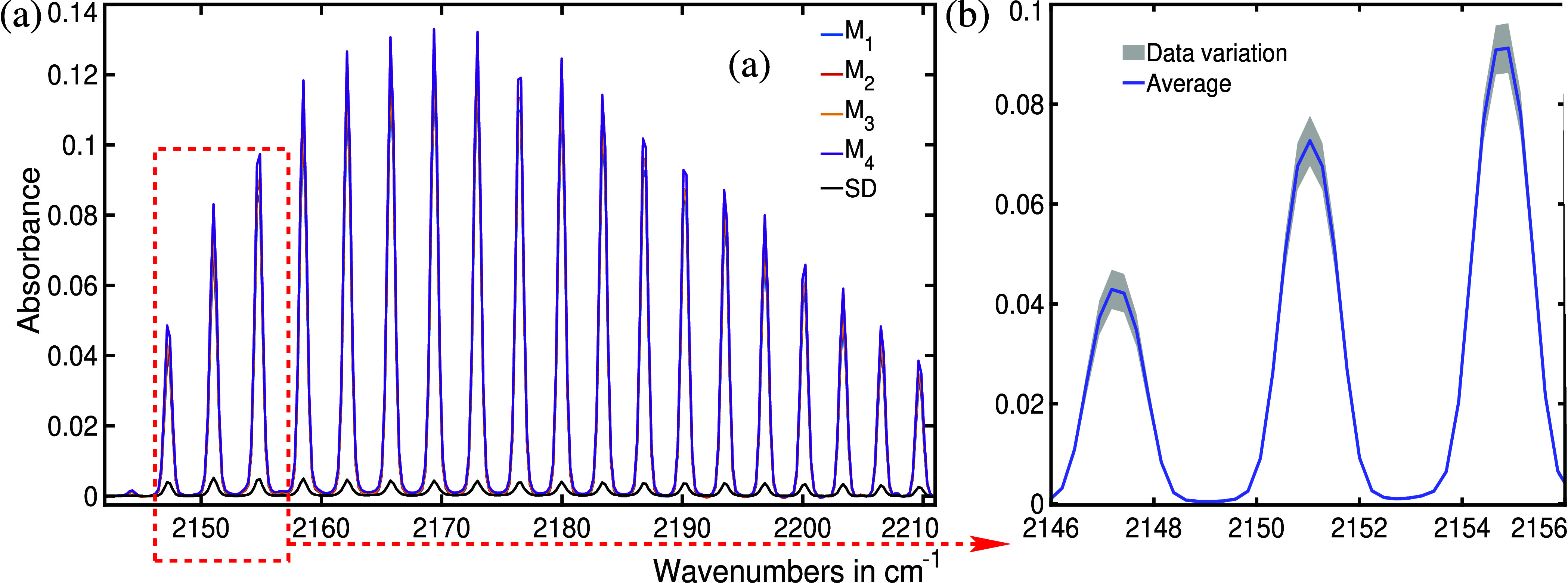
(a) Spectral feature of carbon monoxide (CO) for the headspace of *S. aureus*. *M*_n_ stands for measurement number. The same bacterial species was cultured four times, maintaining the same conditions. In all cases, the measured absorbance in the CO region is nearly identical, indicating a similar population growth of bacteria in all four sample bottles. The shaded area in (b) indicates the standard deviation σ over four measurements.

The preceding analysis and discussion demonstrate that bacteria can be effectively identified by examining their spectral features and their absorption strengths. As absorbance plays a crucial role in bacterial identification via infrared spectroscopy, a pertinent query arises: “How reliable is this parameter?” To address this inquiry, bacteria designated as B01 to B04 were cultured three times each, and B05 was cultured four times under consistent conditions. Each bacterial type exhibited consistent spectral features with nearly identical absorbance signals. For instance, the spectral feature of CO is illustrated for all four headspace measurements of *S. aureus* in Figure 5a. The spectra overlap almost perfectly, indicating consistent CO production across all four of the bacterial cultures. To provide a clear view, this spectral region is further magnified and shown in Figure 5b. In Figure 5b, the average of the four measurements is plotted in blue. The shaded area depicts the calculated standard deviation σ as defined by
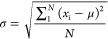
1where *x*_*i*_ is the absorbance at the *i*^th^ spectral position for each spectrum, μ is the average of all data points at each spectral position, and *N* is the total number of data points at each spectral position. The depicted low (σ) value demonstrates the excellent reliability of bacterial identification through infrared spectroscopy.

**Figure 6:**
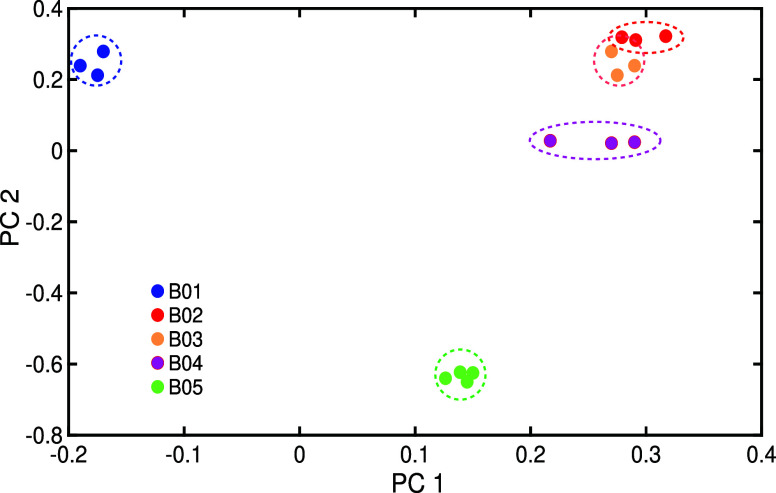
Illustration of blind PCA in a selected spectral region of infrared spectra of the bacterial headspace.

A conventional statistical analysis was performed on the spectral data within the selected region 2140–2210 cm^–1^ using principal component analysis (PCA). The results of this analysis are shown in Figure 6. The data from each individual bacterium formed distinct clusters, which were well-separated from one another. The clustering of the data demonstrates the reproducibility of the bacterial replicas, while the clear separation of these clusters confirms the selectivity of the bacteria.

**Figure 7:**
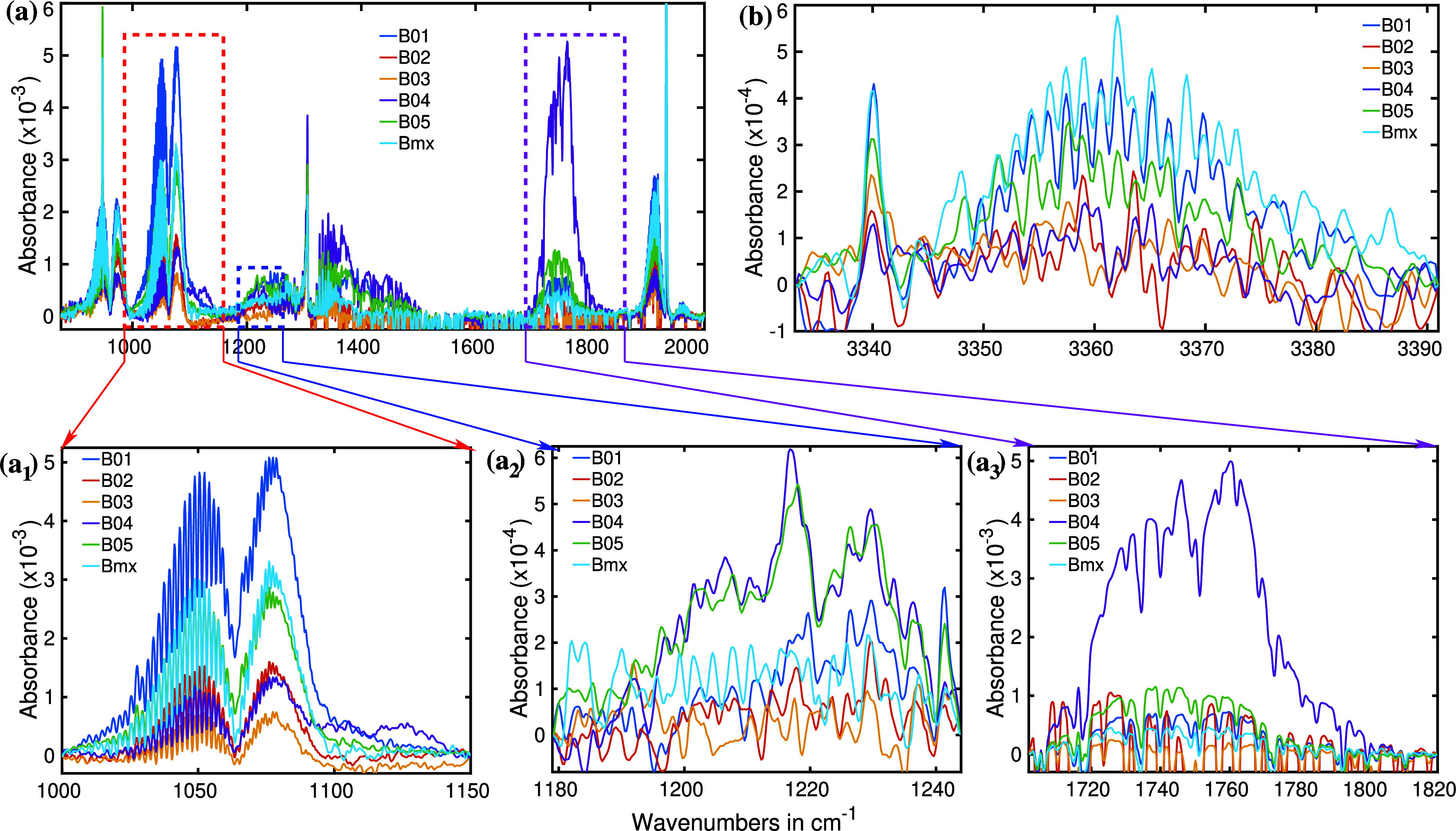
Bacterial headspace spectra of five bacteria individually and as a mixture. B01: *E. coli*, B02: *S. epidemidis*, B03: *P. aeruginosa*, B04: *E. faecalis*, B05: *S. aureus*. Bmx: mixture of B01–B05. (a) spectra at the fingerprint region, (b) N–H stretch vibrational spectral region, (a_1_) CO_2_ spectral signature, (a_2_) spectral signature of acetone, and (a_3_) spectral region of amide-I.

Up to this point, the focus has been on examining the infrared spectral characteristics of an individual bacterial headspace. Yet, in practical scenarios, multiple pathogens coexist within a host body, leading to an anticipated blend of contributions. How efficiently can infrared spectroscopy untangle these varied pathogenic influences? To address this question, several bacteria were cultured together in one flask simultaneously. In Figure 7, the infrared spectra of this mixed bacterial culture alongside spectra obtained from monocultured bacterial headspace are depicted. The spectra corresponding to the mixed bacterial culture are denoted as Bmx.

Figure 7a displays IR absorption spectra in the molecular fingerprint region. The light blue curve illustrates the headspace spectra for mixed bacterial samples consisting of *E. coli*, *S. epidermidis*, *P. aeruginosa*, *E. faecalis,* and *S. aureus*. The multibacterial samples were cultured three times to verify the consistency of the mixed bacterial (Bmx) breath. It is noteworthy that all three absorption spectra are nearly identical. Therefore, only the average spectra are presented here. For a clearer insight into the spectral characteristics, different spectral features are depicted in sub Figures 7a_1_–6a_3_.

In Figure 7a_1_, the absorption spectra are predominantly influenced by carbon dioxide. As previously mentioned, various bacteria generate varying amounts of CO_2_. Consequently, the combined bacterial environment exhibits a pronounced CO_2_ absorption. The mixed bacterial culture produces approximately 60% of the CO_2_ compared to *E. coli* alone, indicating reduced growth in the mixture compared to *E. coli* by itself. This lower growth rate in the bacterial mixture may be due to competition among different bacterial species. However, pinpointing the exact contributions of each bacterial species to the observed CO_2_ absorption peak is challenging because CO_2_ production is a common feature of bacterial metabolism. Moreover, prior discourse revealed that different bacteria yield distinct metabolites during their metabolic activities. Hence, exploring additional absorption characteristics becomes imperative.

As discussed previously, investigations revealed that two of five selected bacteria generate acetone during their metabolic processes. Therefore, analyzing the spectral characteristics of acetone could offer further insights. In Figure 7a_2_, a magnification of around 1215 cm^–1^ is depicted. Notably, the light blue curve exhibits no discernible acetone fingerprint. Consequently, one can conclude that *E. faecalis* and *S. aureus*, both known to produce significant amounts of acetone in their metabolic pathways, do not contribute to the observed spectra. This outcome is not unexpected given the competitive dynamics within a bacterial mixture, where certain strains may struggle to proliferate. It is plausible that *E. faecalis* and *S. aureus* either failed to grow or experienced minimal population growth, rendering them undetectable by the current detection methods.

Further support for the aforementioned argument is gleaned by examining the amide-I spectral feature, which is predominantly absorption due to the C=O stretch vibration. The amide-I spectral region is magnified and depicted in Figure 7a_3_ to enhance clarity. If the population growth of *E. faecalis* was normal, then a pronounced absorption peak would be anticipated in the light blue curve. However, the light blue spectra exhibit minimal elevation in the amide-I band. We performed a multivariate spectral analysis
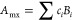
2where *A*_mx_ is the absorption strength of a spectral band of the bacterial mixture, *B*_*i*_ is the absorbance of the same band for the individual (*i*^th^) bacterium, and *c*_*i*_ is the absorption coefficient for *i*^th^ bacterium in the mixture. Analyzing the spectral intensity of the individual and the mixture of bacteria at the amide-I band, we calculated *c*_*i*_ = 0.61 for *E. coli*. The coefficient for all other bacteria is zero. This is also well matched with the corresponding CO_2_ absorption strength. Therefore, it can be concluded that in the bacterial competition, only *E. coli* survives and thrives; however, due to the competition, the growth of *E. coli* is lower than its natural growth.^59,60^

It is likely that *E. coli* is the predominant pathogen in the mixed bacterial culture. Further supporting evidence is seen in the spectral region around 3360 cm^–1^, characteristic of the amide-A band, primarily indicating N–H stretch vibration.^61^ A reasonably strong and broad peak is observed for the light blue curve, which is comparable in strength to the absorption peak of *E. coli*. This peak serves to confirm that the *E. coli* population is the highest within the bacterial mixture.

Based on the aforementioned observation, it can be inferred that bacteria can be distinguished by examining their volatile metabolites through infrared spectroscopy. Even when multiple bacteria coexist, identification is feasible, provided that their populations are adequate, given knowledge of their individual metabolic profiles. Adopting this method can potentially enhance the diagnostics of resident pathogenic bacteria that are challenging to culture. For instance, *Helicobacter pylori*, a common pathogen in the human stomach, can typically only be cultured from biopsies, a process prone to failure due to the demanding culture requirements of the bacterium. Albeit a *Helicobacter pylori* diagnostic breath test already exists, it is solely based on urea breakdown and does not provide identification of involved bacteria. Similarly, *Clostridioides difficile*, which is another bacterium responsible for severe gastrointestinal conditions, presents diagnostic challenges. Some effort was already made to identify unique VOCs in breath, plasma, and stool samples from patients with *C. difficile*.^62^ If those bacteria could be rapidly and accurately detected in human breath, this would be a milestone in the diagnostics of pathogenic bacteria.^13^

## 4 Conclusions

This article presents an investigation of bacterial breath using infrared spectroscopy. We examined five distinct bacterial strains, individually and in combination, by growing them and collecting their headspaces for the analysis of volatile metabolites via infrared spectroscopy. This study has successfully identified numerous volatile metabolites generated through metabolic processes. While certain metabolites are common to all bacteria, it appears that each bacterial strain also has distinct metabolites that can be identified. Furthermore, concentrations of common metabolites exhibit considerable variation. These distinctive behaviors enable the differentiation of bacteria within mixed bacterial environments. This methodology holds promise for developing rapid diagnostics free from the need for bacterial culture, providing thorough characterization of individual bacterial strains. Ongoing development includes the bacterial growth dynamics and the development of an infrared spectral database cataloging bacterial headspace profiles.

## Supporting Information Available

Reproducibility of bacterial cultures demonstrated by presenting examples of all four bacteria across two different spectral regions (PDF)
