# Supplementary material for: Measurement of Bacterial Headspaces by FT-IR Spectroscopy Reveals Distinct Volatile Organic Compound Signatures
Source: Anal Chem. 2024 Dec 21;97(1):106–13. doi: 10.1021/acs.analchem.4c02899 (PMC11740187; doi:10.1021/acs.analchem.4c02899)
Supplement: Supplementary file 1 — ac4c02899_si_001.pdf [file ac4c02899_si_001.pdf]

Supporting Information
Measurement of bacterial headspaces by FT-IR spectroscopy reveals distinct volatile organic compound signatures

Christian Zenner[1], Lindsay J Hall[1,2], Susmita Roy[3], Jürgen Hauer[4], Ronald Sroka[5,6], and Kiran Sankar Maiti[4,6,*]

[1]Technical University of Munich, School of Life Sciences, Intestinal Microbiome, Weihenstephaner Berg 3, 85354 Freising, Germany
[2]University of Birmingham, Institute of Microbiology and Infection, Chair of Microbiome Research, B15 2TT Edgbaston Birmingham, United Kingdom
[3]Technical University of Munich, School of Medicine and Health, Department of Clinical Medicine, Klinikum rechts der Isar, Ismaninger Str. 22, 81675 Munich, Germany
[4]TUM School of Natural Sciences, Department of Chemistry, Technical University of Munich, 85748 Garching, Germany
[5]Department of Urology, LMU University Hospital, LMU Munich, 81377 Munich, Germany
[6]Laser-Forschungslabor, LIFE-Center, LMU University Hospital, LMU Munich, 82152 Planegg, Germany
[*]Corresponding author: kiran.maiti@tum.de

Contents

1 Introduction **S2**

2 CO absorption spectra **S2**

3 CH_4 absorption spectra **S4**

1 Introduction

To establish the utility of infrared spectroscopy for bacterial identification via headspace analysis, it is crucial to ensure the reproducibility of bacterial cultures[1, 2, 3]. In our experiment, we selected five bacterial strains: *Escherichia coli* WS 1322 (B01), *Staphylococcus epidermidis* WS 4374 (B02), *Pseudomonas aeruginosa* DSM 19880 (B03), *Enterococcus faecalis* DSM 20371 (B04), and *Staphylococcus aureus* WS 228 (B05). To guarantee reliability, the first four bacterial strains were replicated three times in separate sample bottles, while the fifth strain was replicated four times. To demonstrate the reproducibility of bacterial cultures, the spectral feature of carbon monoxide is highlighted in the main article. All the data are analyzed using well developed SOP of breath analysis[4, 5, 6, 7]. Figures S1–S4 present the CO spectra for the bacteria B01–B04. In general, each replicate for the corresponding bacterial strain exhibited excellent reproducibility. The data deviations are indicated as shaded areas in the right panel of each plot.

2 CO absorption spectra

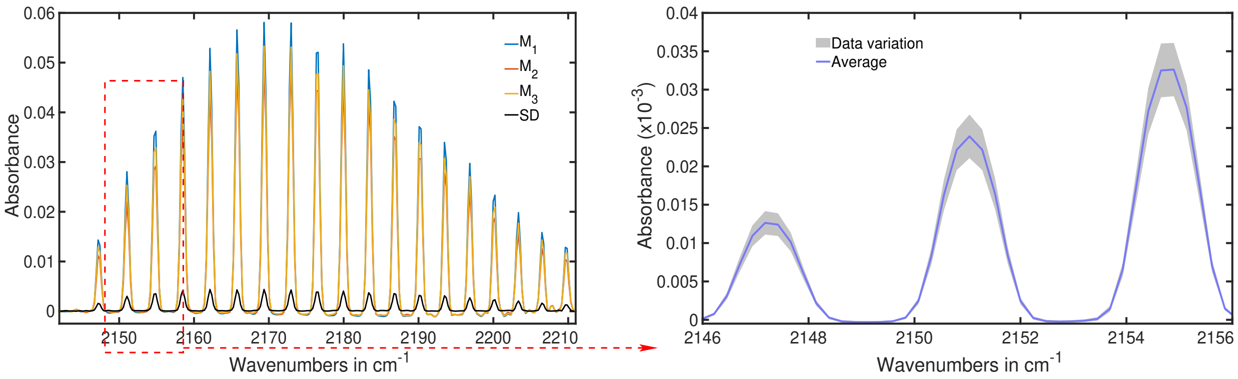

Figure S1: Left side plot: Spectral feature of carbon monoxide (CO) for headspace of *Escherichia coli* WS 1322 (B01). M_n stands for the measurement number. The same bacterial species was cultured three times, maintaining the same conditions. In all cases, the measured absorbance in the CO region is nearly identical, indicating a similar population growth of bacteria in all three sample bottles. Right side plot: The shaded area indicates the standard deviation σ over three measurements.

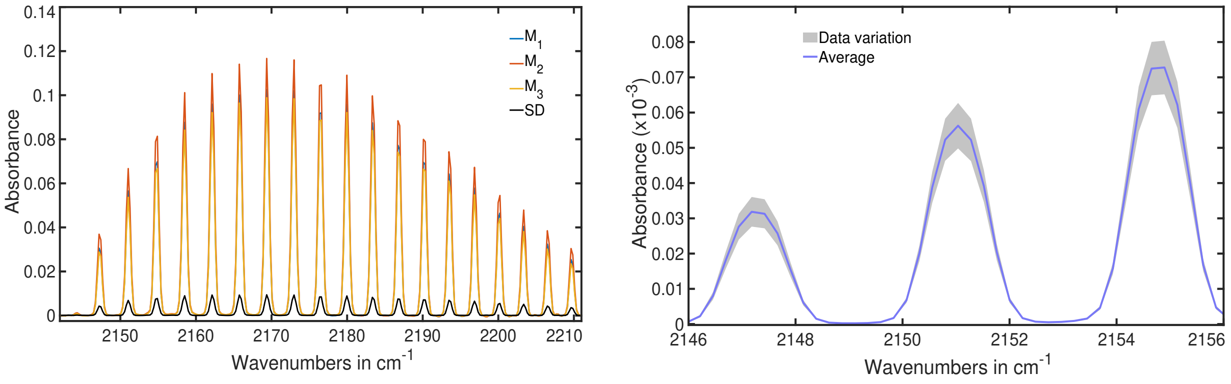

Figure S2: Left side plot: Spectral feature of carbon monoxide (CO) for headspace of *Staphylococcus epidermidis* WS 4374 (B02).

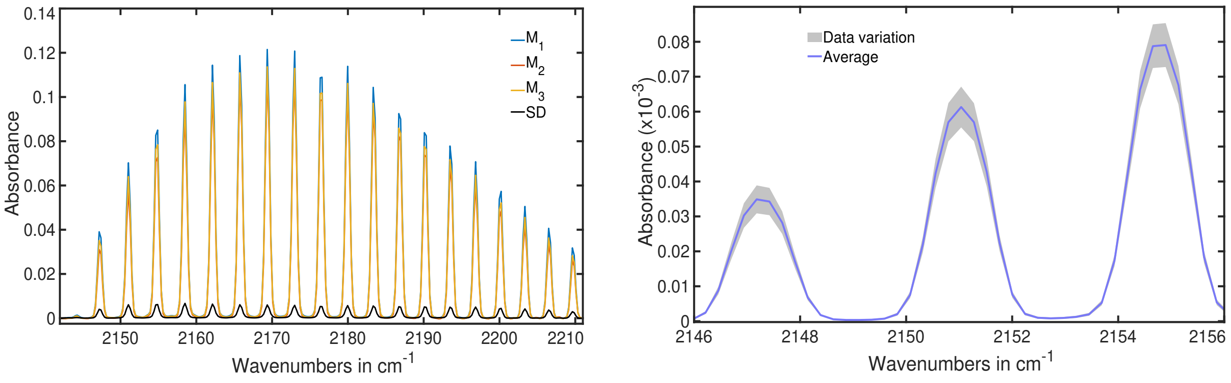

Figure S3: Left side plot: Spectral feature of carbon monoxide (CO) for headspace of *Pseudomonas aeruginosa* DSM 19880 (B03).

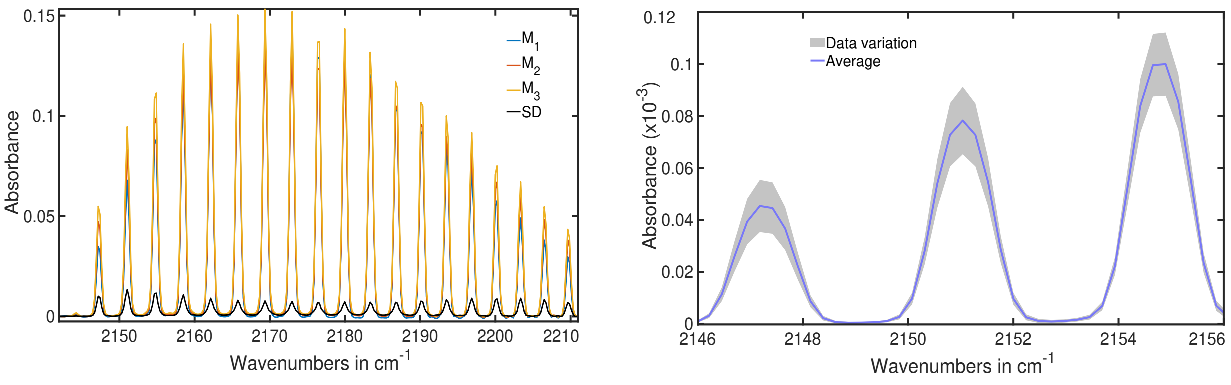

Figure S4: Left side plot: Spectral feature of carbon monoxide (CO) for headspace of *Enterococcus faecalis* DSM 20371 (B04).

3 CH_4 absorption spectra

As supporting evidence, we also plotted the spectral features of methane (CH_4) observed in the spectral region around 3000 cm^{-1}, as shown in Fig. S5–S9. In the left panel of each figure, the absorption spectra for individual headspace samples from bacterial replicates are displayed, along with their standard deviation (black line). In the right panel, the average spectra are plotted with a blue line, while the spectral range is represented by a gray shaded area.

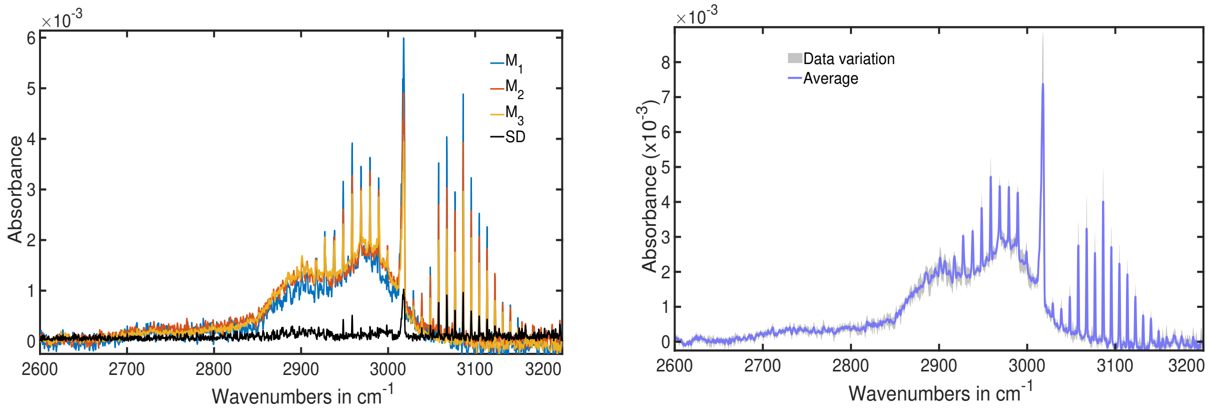

Figure S5: Left side plot: Spectral feature of methane (CH_4) for headspace of *Escherichia coli* WS 1322 (B01).

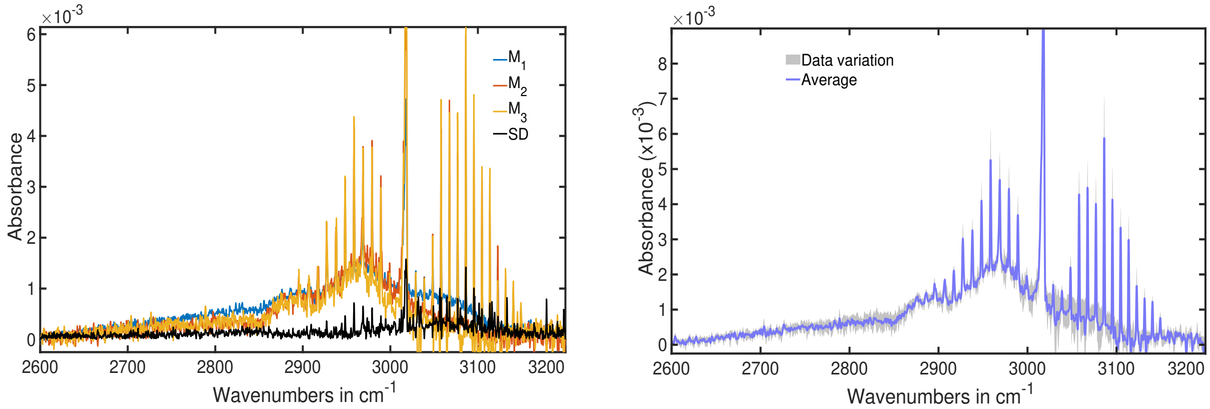

Figure S6: Left side plot: Spectral feature of methane (CH_4) for headspace of *Staphylococcus epidermidis* WS 4374 (B02).

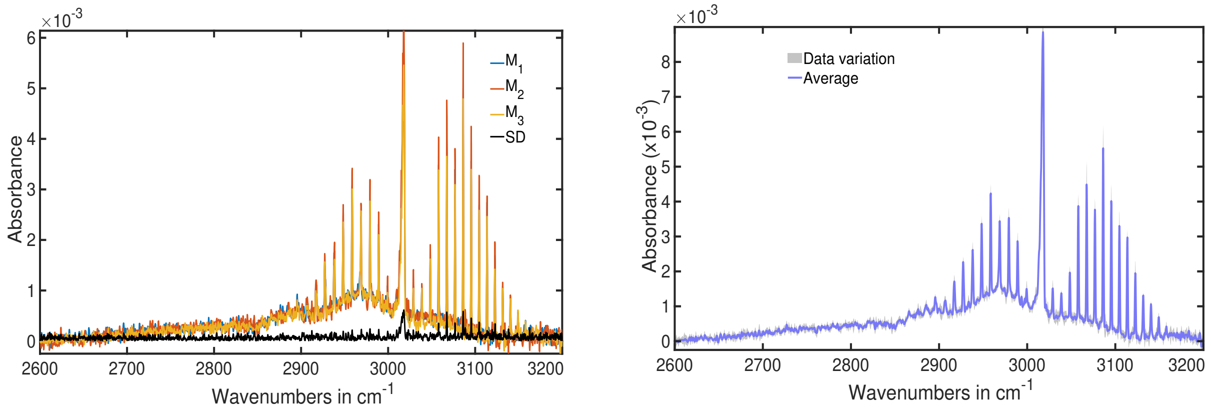

Figure S7: Left side plot: Spectral feature of methane (CH_4) for headspace of *Pseudomonas aeruginosa* DSM 19880 (B03).

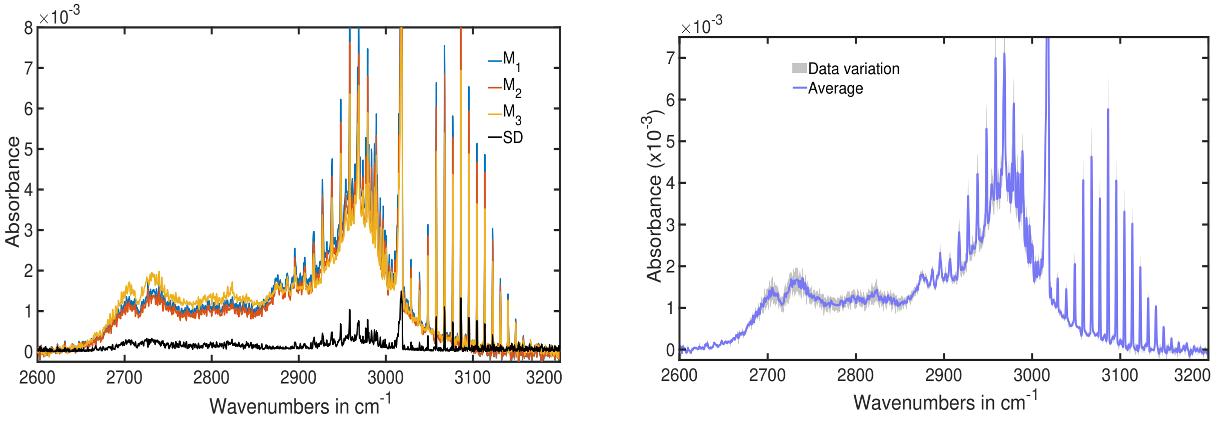

Figure S8: Left side plot: Spectral feature of methane (CH_4) for headspace of *Enterococcus faecalis* DSM 20371 (B04).

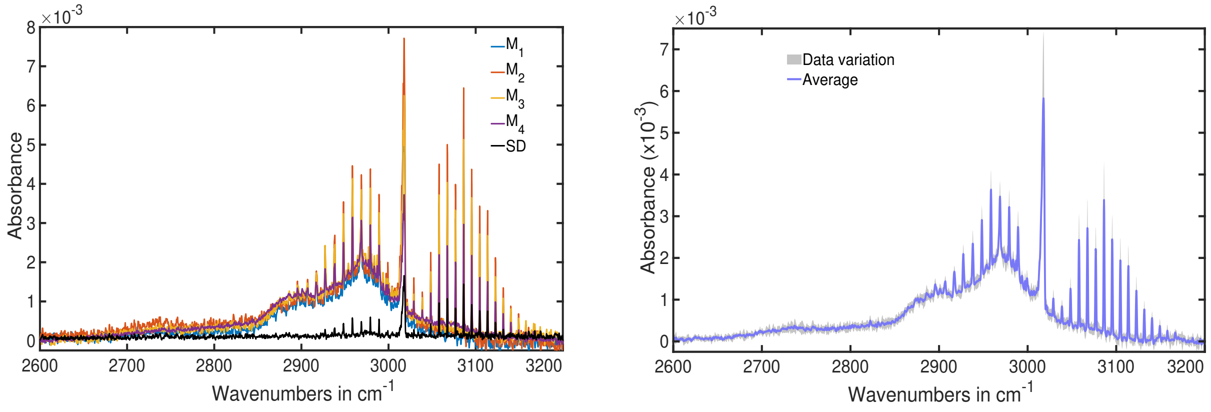

Figure S9: Left side plot: Spectral feature of methane (CH_4) for headspace of *Staphylococcus aureus* WS 228 (B05).

References

[1] Kiran Sankar Maiti, Michael Lewton, Ernst Fill, and Alexander Apolonski. Sensitive spectroscopic breath analysis by water condensation. *Journal of Breath Research*, 12(4):046003, 2018.

[2] Kiran Sankar Maiti and Alexander Apolonski. Monitoring the reaction of the body state to antibiotic treatment against helicobacter pylori via infrared spectroscopy: A case study. *Molecules*, 26(11), 2021.

[3] Nadia Feddahi, Lea Hartmann, Ursula Felderhoff-Müser, Susmita Roy, and et.al. Neonatal exhaled breath sampling for infrared spectroscopy: Biomarker analysis. *ACS Omega*, 9(28):30625–30635, July 2024.

[4] Alexander Apolonski, Susmita Roy, Renée Lampe, and Kiran Sankar Maiti. Molecular identification of bio-fluids in gas phase using infrared spectroscopy. *Appl. Opt.*, 59(17):E36–E41, Jun 2020.

[5] Alexander Apolonski and Kiran Sankar Maiti. Towards a standard operating procedure for revealing hidden volatile organic compounds in breath: the Fourier-transform IR spectroscopy case. *Appl. Opt.*, 60(14):4217–4224, May 2021.

[6] Susmita Roy and Kiran Sankar Maiti. Baseline correction for the infrared spectra of exhaled breath. *Spectrochimica Acta Part A: Molecular and Biomolecular Spectroscopy*, page 124473, 2024.

[7] Maxim F. Gelin, Alexander P. Blokhin, Elena Ostrozhenkova, Alexander Apolonski, and et.al. Theory helps experiment to reveal VOCs in human breath. *Spectrochimica Acta Part A: Molecular and Biomolecular Spectroscopy*, page 119785, 2021.
